# Ambulatory Catheter-based Interscalene Block for Proximal Humerus Fracture Rehabilitation: Safety, Efficacy and Lessons from a Pilot Study

**DOI:** 10.5704/MOJ.2507.011

**Published:** 2025-07

**Authors:** MFK Nah, ZQ Seng, YJB Tan

**Affiliations:** 1 Department of Orthopaedic Surgery, Tan Tock Seng Hospital, Singapore; 2 Department of Anaesthesiology, Woodlands Health Campus, Singapore; 3 Department of Orthopaedic Surgery, Woodlands Health Campus, Singapore

**Keywords:** orthopaedic surgery, nerve block catheters, post-operative pain, proximal humerus fracture

## Abstract

**Introduction:**

Proximal humerus fractures (PHFs) are associated with morbidity/functional impairment. Rehabilitation adherence is crucial to regain independent function yet is often hindered by pain. This pilot study aims to analyse the safety and efficacy of ambulatory catheter-based interscalene blocks (CISBs) as analgesia in post-surgical PHF patients and summarise learning points to guide further implementation/study of ambulatory CISB.

**Materials and methods:**

This pilot study selected PHF patients who were >18yo, surgically treated and received ambulatory CISB (CISB ≥72 hours). Data was derived from clinical documentation (anaesthetist/surgeon/therapist reviews). Clinical outcomes (e.g. range of motion, Quick Disability of Arm/Shoulder/Hand (qDASH) scores), dynamic/resting pain scores and incidence of CISB-related complications were collected.

**Results:**

Twelve patients were selected with mean ambulatory CISB duration of 9.5 days. All patients improved clinically, with means improvements of +64.6° and +61.9° for passive flexion and abduction, and reduction of 29.8 in qDASH after 3 months. Two patients experienced neurological complications (phrenic nerve palsy; medial forearm numbness) while six patients experienced catheter-based complications (dislodgment, erythema). All complications were self-limiting, resolving with removal of catheter.

**Conclusion:**

Ambulatory CISB can minimise pain and facilitate rehabilitation for PHF patients. Learning points include (1) complications are predictable and incidence/physiological impact on patients can be minimised via appropriate patient selection, (2) standardised protocols (e.g. tunnelling of catheters) help maximise utility of ambulatory CISB while minimising complications, (3) regular monitoring/anticipation of complications facilitate early detection and prompt management. These learning points, combined with existing literature, can be adapted to future applications of ambulatory CISB to better study its safety and efficacy.

## Introduction

The proximal humerus fracture (PHF) is the 3rd most common non-axial osteoporotic fracture, 3rd only to hip and distal radius fractures in elderly patients^1^. With the rapid development of various surgical implant options (e.g. locking plate-screw constructs, reverse shoulder arthroplasty) for the treatment of PHFs, a growing number of surgeons are opting for operative management for PHFs they might otherwise have managed conservatively in the past, especially for physiologically younger patients^2-5^. Postoperatively, early shoulder mobilisation has also been shown to improve functional outcomes in terms of range of motion (ROM), pain scores, etc. – without feared complications such as displacement/non-union^6-8^.

A recurring theme in the literature was that pain during exercise is often the most commonly patient-reported and therapist-reported barrier to rehabilitation adherence^9^. Existing post-operative analgesia modalities include oral, parental, nerve-block techniques (single-shot or catheter-based techniques) or even intraarticular analgesia^10^. Notably, in a large-scale systemic review by Iliaens *et al*, regional anaesthesia (specifically in the form of an interscalene block) has had promising results in PHF surgery rehabilitation: decreasing opioid requirements and improving upper limb function (e.g. Quick Disabilities of the Arm, Shoulder and Hand (quickDASH) scores) and shoulder range of motion^11^. Different centres have adopted different modalities of regional anaesthesia for shoulder surgery, such as single-shot interscalene block (SISB) which provides effective analgesia up to 8 hours post-operatively, SISB with adjuvant agents (e.g. dexamethsone) to prolong duration of effective analgesia up to 24-72 hours or continuous catheter-based interscalene blocks (CISBs) which can remain insitu up to 72 hours^11,12^.

Poorly controlled pain has been shown to be inhibitory to rehabilitation compliance in the early rehabilitation stage and hence lead to poorer functional outcomes^13^. The study team proposed that CISBs, when used for 72 hours or longer (“ambulatory CISB”) can be a safe form of post-operative analgesia for patients undergoing surgical treatment of PHFs, extending analgesia beyond the post-operative phase into the initial rehabilitation phase, improving rehabilitation compliance and patient outcomes.

Ambulatory CISB was piloted within our institution and this study aims to analyse its safety profile and efficacy and determine if this novel application of existing analgesia deserves to be studied further.

## Materials and Methods

The aim of this pilot study is to determine the safety and efficacy of ambulatory CISB as an analgesia option in post-surgical rehabilitation of PHFs. The author’s institution’s domain specific review board approved this study and its methodology.

For study population, the study team recruited patients from a 12-month period from June 2020 to June 2021 based on the following inclusion criteria (Table I). Patients were prospectively selected if they were above 18 years old, opted for surgical management of PHF, and received ambulatory CISB (CISB duration ≥72 hours).

**Table I:** Inclusion criteria used to select patients.

Inclusion Criteria for Study
1. Above 18 years of age
2. Surgically treated proximal humerus fractures
3. Ambulatory catheter-based interscalene block (CISB for 72 hours or more)

For data collection, the patients were followed-up closely by a multi-disciplinary team – which consisted of the orthopaedic surgery team, acute pain service team and occupational therapists. Patients attended the orthopaedic surgery clinic at post-operative two week-, one month-, three months-mark and subsequently as required for review of surgical wound site, symptomology (pain, etc.) and function. The acute pain service which performed the CISB procedure would also regularly follow patients up while the CISB was in-situ for CISB-related complications. This was done via daily telemedicine consultations with review of clinical photographs taken by patients, as well as weekly clinic visits. Lastly, patients also attended regular occupational therapy sessions for standardised rehabilitation exercises and functional scoring such as range of motion measurements, quick Disabilities of Arm/Shoulder/Hand (quickDASH) score. Clinical documentation from these multi-disciplinary follow-ups were recorded on our institution’s electronic medical records and then reviewed for pre-determined outcome measures, which included functional measures (range of shoulder flexion and abduction, quickDASH score), pain measures (numerical pain rating scale (NPRS) score during rest and during dynamic movements) as well as any documented complications due to CISB.

Complications of CISB are divided into mechanical complications (arising from insertion of catheter or having a foreign body in-situ) and neurological complications (Table II). Mechanical complications include dislodgement, soft tissue infections and injury to locoregional structures (pneumothorax/hemothorax, common carotid/vertebral artery injury), etc. Neurological complications include unintended neural injury/blockade (e.g. phrenic nerve, recurrent laryngeal nerve, sympathetics, brachial plexus) or even local anaesthesia (LA) systemic toxicity, etc.

**Table II:** Non-exhaustive summary of complications of CISB.

Mechanical Complications of CISB		Neurological Complications of CISB
1. Catheter-related		1. Local anesthetic systemic toxicity (LAST)
a. Kinking		2. Peripheral nerve injury
b. Dislodgement / Migration c. Bleeding/haematoma 2. Soft-tissue infection – e.g. cellulitis, abscess, etc.		a. E.g. phrenic nerve, recurrent laryngeal nerve, long thoracic nerve, dorsal scapular nerves, sympathetic trunk, brachial plexus
3. Injury to surrounding structures		
a. E.g. carotid/vertebral artery puncture		
b. E.g. pneumothorax/haemothorax		

**Fig. 1:**
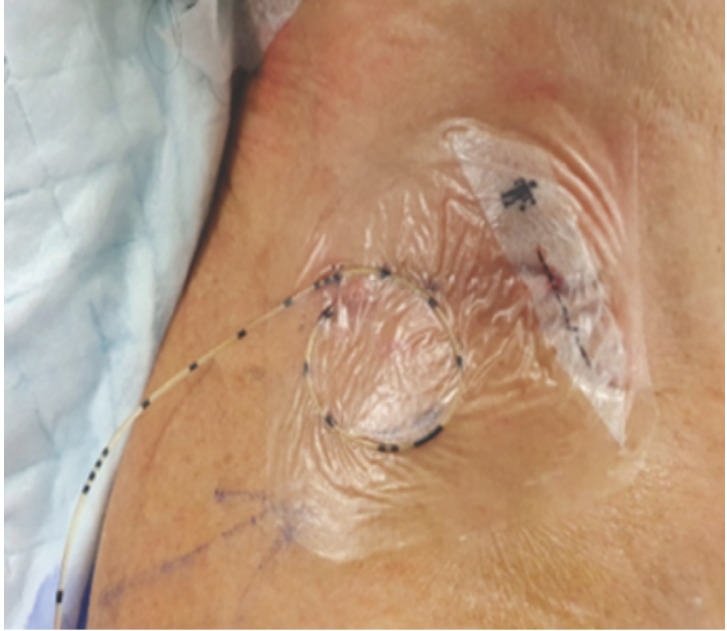
Example of an in-situ catheter-based interscalene block catheter performed in the author’s institution.

For technique of CISB insertion, the interscalene block provides analgesia and surgical anaesthesia to the shoulder and proximal arm (see Fig. 1 for an example of an in-situ interscalene nerve block catheter). Regional anaesthetic agent is inflitrated into the interscalene groove between the anterior and middle scalene muscles to achieve blockade of the brachial plexus at the level of the nerve roots. With this technique, there is reliable blockade to cervical five and cervical six nerve roots which can give good analgesia to lateral clavicle, shoulder joint and proximal humerus. In our institution, the interscalene block is performed with ultrasound guidance, usually a linear probe transversely across the lateral neck at the level of the cricoid cartilage. Important structures such as anatomical landmarks or vital structures to avoid are first sonographically identified, which include, but are not limited to, the subclavian artery/vein, lung pleura, common carotid artery, phrenic nerve and brachial plexus trunks, before introduction of the in-dwelling cathether. On top of the in-dwelling catheter, the CISB also consists of an infusion bulb which holds a reservoir of anaesthetic agent. Arrangements were made for the infusion bulbs to be topped up with the anaesthetic drug during rehabilitation therapy visits, to minimise the number of visits for patients. For patients within this study, the catheters and anaesthetic agent/doses were standardised. The B. Braun Contiplex® S Ultra 50mm catheter sets was inserted out-of-plane under ultrasound guidance, and an initial bolus of 10-15ml of ropivacaine 0.4% was given followed by infusion of ropivacaine 0.2% at 1-3ml per hour, titrated to numbness/patient pain levels.

## Results

Twelve patients (numbered #1 to #12 in chronological order) were selected for this pilot study. This included 7 females and 5 males, with a mean age of 60.2 years (35 – 80). Nine patients underwent open reduction and internal fixation, while three underwent reverse shoulder arthroplasty for treatment of PHF. Patients had a mean duration of 9.5 days (3 – 15) with ambulatory CISB.

Complications in this pilot study are summarised in the Table III. While the complication rate in the sample population was high, with 8 out of 12 patients experiencing some form of mechanical or neurological complication, it should be noted that all complications were self-limiting and did not require further management, short of removing the catheter earlier than planned.

**Table III:** Patient demographics and learning points.

Patient #	Gender	Age	Duration of CISB (days)	Surgery performed	Tunnelled or Non-tunnelled	Mechanical Complications	Neurological Complications	Remarks / Learning Points
1	Female	62	15	ORIF*	Non-tunnelled -> converted to tunnelled on post-op day 2	Dislodgement on post-op day 2	Grade I	Tunnelling is important to prevent dislodgement.
2	Male	61	7	ORIF	Tunnelled	Surrounding erythema noted on post-op day 5	-	Self-limiting erythema which resolved with removal of CISB catheter. Patient selection is important: patient had multiple comorbidities including poorly controlled diabetes - which likely predisposed to soft tissue infection.
3	Female	80	16	RSA#	Non-tunnelled -> converted to tunnelled on postop day 2	Dislodgement on post-op day 2	-	Tunnelling is important to prevent dislodgement. Presence of CISB raises more diagnostic dilemmas: Patient experienced shortness of breath with tachycardia - this was eventually attributed to type 2 myocardial infarction from operative stress / pulmonary embolism. Presence of CISB raised diagnostic dilemmas as phrenic nerve palsy secondary to CISB was also a consideration.
4	Male	35	3	ORIF	Tunnelled	-	-	
5	Female	64	5	RSA	Tunnelled	-	Phrenic nerve palsy	Patient selection is important: this patient was diagnosed with hemidiaphragm paralysis secondary to right phrenic nerve paresis. As patient was fit with no cardiorespiratory co-morbidities, this complication was self- limiting.
6	Male	59	7	ORIF	Tunnelled	Dislodgement on post-op day 6		Patient education is paramount for the success of CISB. This complication can be considered as a false positive as it was revealed that patient had manipulated the catheter to test if he could cope with rehabilitation without the analgesia from the CISB.
7	Female	63	7	ORIF	Tunnelled	-	-	
8	Male	55	8	ORIF	Tunnelled	-	-	
9	Female	61	4	RSA	Tunnelled	-	-	Presence of CISB raises more diagnostic dilemmas: Patient experienced wrist drop post-operatively. This was eventually attributed to intra-operative neuropraxia rather than an inadvertent neurological complication from CISB (proven as wrist drop persisted even when infusion of anaesthetic agent was stopped)
10	Female	73	15	ORIF	Tunnelled	Surrounding erythema noted on post-op day 16		Similar to other in-situ instrumentation, there should be a cut-off duration for CISB to prevent soft tissue infection and complications. Erythema was self-limiting and resolved with removal of CISB catheter.
11	Female	53	14	ORIF	Tunnelled		Persistent medial forearm numbness	Neurological symptoms were attributed to the CISB as the deficits resolved with removal of CISB.
12	Male	56	13	ORIF	Tunnelled	Dislodgment on post-op day 12	-	

Two patients experienced neurological complications. Patient #5 was a 64-year-old Malay lady who had underwent reverse shoulder arthroplasty for a right PHF in June 2020 and presented with dyspnea and desaturation to 88% on room air on post-operative day 2. This was eventually attributed to right phrenic nerve palsy, as evidenced by radiographic features of right middle and lower lung lobe collapse. Her condition was treated by lowering the rate of local anaesthetic infusion (and subsequent removal of CISB prior to discharge), incentive spirometry and supportive treatment. She improved promptly, being discharged on postoperative day 4 after CISB was removed. Patient #11 was a 53-year-old Chinese lady who underwent open reduction internal fixation for a right PHF in January 2021. Likewise, she had a tunneled CISB performed. This lady experienced self-limiting medial forearm numbness in the distribution of the medial cutaneous nerve of the forearm that resolved promptly after removal of the CISB catheter.

Another six patients experienced catheter-based complications. Four patients experienced catheter displacement, while two patients experienced catheter site erythema which was self-limiting and did not require additional treatment (e.g. antibiotics). The four patients who experienced catheter displacement were patients #1 (62-year-old Chinese female undergoing ORIF for right PHF in June 2020), #3 (80-year-old Indian female undergoing RSA for right PHF in June 2020), #6 (59 year old Chinese male undergoing ORIF for left PHF in July 2020) and #12 (56 year old Malay male undergoing ORIF for left PHF in March 2021). Patients #1 and #3 were the only patients in our series who had non-tunneled CISBs inserted (as they were both performed by the same anaesthetist who was unfamiliar with the tunneled technique). Their CISB was repeated subsequently with tunneling of the catheter by another anaesthetist and they did not experience further issues with dislodgement. Patients #6 and #12 had tunneled CISBs from the beginning. Patient #6 was noted to have his catheter dislodged on post-operative day 6 during teleconsultation with the pain team. On further questioning, this gentleman had intentionally manipulated his catheter to see if he was able to bear with rehabilitation without the CISB, under the impression that he would be able to easily re-insert the CISB to its original position if unable to bear with the pain. Patient #12 experienced slippage of his CISB by 3cm at postoperative day 12 and decision was made for complete removal as the CISB was only providing analgesia to the cervical five dermatome. Two patients experienced catheter-site erythema which resolved with removal of the catheter – none of these patients required additional treatment (e.g. surgical debridement, antibiotics). Patient #2 was a gentleman with poorly controlled diabetes who underwent ORIF for his left PHF in May 2020 and experienced catheter-site erythema on post-operative day 5 – this resolved once the catheter was removed. Patient #10 (73-year-old lady undergoing ORIF for right PHF in November 2020) experienced mild erythema around catheter site on post-operative day 16 that resolved once the catheter was removed as well.

For clinical outcomes, in terms of shoulder function, there was improvement in mean clinical outcome measures (passive shoulder flexion, passive shoulder abduction, qDASH) during the three-month rehabilitation period (Table IV). Although the sample size of this pilot study was not significant enough for meaningful statistical analysis, all 12 patients included in this study saw improvements temporally from post-operative measurements to 3-month follow-up. All but 2 patients (patient #9 and patient #12) were able to achieve functional shoulder flexion/abduction ROM (shoulder ROM required to independently carry out activities of daily living – established to be 115° flexion / 120° abduction^14^) by the 3-month post-operative mark.

**Table IV:** Shoulder function outcomes measures at different time-points.

Mean Clinical Outcome Measures	Post-operatively	Prior to Removal of CISB	At three-month follow-up
Passive Shoulder Flexion (°)	60.0 (30-90)	78.3 (45-125)	124.6 (110-150)
Passive Shoulder Abduction (°)	57.1 (45-90)	79.8 (45-128)	119.0 (95-135)
qDASH score	52.2 (25-67.5)	-*	22.4 (2.5-56.8)

Note: *qDASH score was only re-assessed at three-month post-operative follow-up

In terms of pain score, patients had higher mean rest and pain scores with CISB removed compared to when they had CISB in-situ (Table V). Further, a paired samples t-test was conducted to compare pain score at the latest time-point with the CISB insitu and earliest time-point with CISB removed (Table VI). There was a no significant difference in the mean resting NPRS scores with CISB situ and mean resting NPRS scores with CISB removed (t(11) = -0.220, 2-tailed sig = 0.830). However, there was a significant difference in the mean dynamic NPRS scores with CISB situ and mean dynamic NPRS scores with CISB removed (t(11) = -3.761, 2-tailed sig. = 0.003).

**Table V:** Mean rest/dynamic pain scores with CISB in-situ and removed.

	Mean (SD)	Range
Rest NPRS with CISB in-situ	0.917 (1.73)	0-5
Rest NPRS with CISB removed	1.00 (2.13)	0-6
Dynamic NPRS with CISB in-situ	2.75 (2.49)	0-6
Dynamic NPRS with CISB removed	4.25 (2.22)	1-8

**Table VI:** Paired t-test comparison of mean rest/dynamic pain score with CISB in-situ and CISB removed.

	Paired Samples Test					t	df	Sig. (2-tailed)
	Paired Differences							
	Mean	Std. Deviation	Std. Error Mean	95% Confidence Interval of the Difference Lower Upper				
				Lower	Upper			
Rest NPRS with CISB in-situ - Rest NPRS with CISB removed	-.08333	1.31137	.37856	-.91654	.74987	-.220	11	.830
Dynamic NPRS with CISB in-situ - Dynamic NRPS with CISB removed	-1.50000	1.38170	.39886	-2.37789	-.62211	-3.761	11	.003

When comparing between type of surgery performed (ORIF vs RSA), it was found that there was a significant difference in dynamic NPRS with CISB insitu depending on type of surgery performed (p = 0.018) (Table VII).

**Table VII:** 2-tailed T-test comparison of surgery type (open reduction internal fixation vs reverse shoulder arthroplasty) with clinical outcome measures.

		Clinical Outcome Measures				Rest NPRS with CISB removed	Dynamic NPRS with CISB in-situ	Dynamic NPRS with CISB removed
		Flexion PROM at 3-month	Abduction PROM at 3-month	DASH Score at 3-month	Rest NPRS with CISB in-situ			
Mean values (SD)	ORIF	122.78	118.11	29	1.22	1.22	3.67	4.56
		(σ = 10.93)	(σ = 11.97)	(σ = 19.61)	(σ = 1.92)	(σ = 2.44)	(σ = 2.18)	(σ = 2.51)
	RSA	130	130	12.13	0	0.33	0	3.33
		(σ = 17.32)	(σ = 12.58)	(σ = 13.32)	(σ = 0)	(σ = 0.58)	(σ = 0)	(σ = 0.58)
p-value (2-tail T test)		0.40543	0.668697	0.208819	0.31131	0.557268	0.018116	0.435311

## Discussion

This pilot study has demonstrated that ambulatory CISB can be a viable option of analgesia for PHF patients to significantly reduce dynamic pain in the immediate 72 hours post-operative phase as well as the early rehabilitation stage past 72 hours – it deserves more attention and should be studied further. Patients in this study saw 0.083 points lower (-8.3%) mean resting NPRS with CISB analgesia and 1.50 points lower (-35.3%) mean dynamic NPRS with CISB analgesia. CISB can help to achieve dynamic NPRS reduction greater than the established minimal clinically important difference (MCID) of 29%^15^. The ability of ambulatory CISB to provide on-demand analgesia during timepoints of expected increased in dynamic pain (e.g. therapy sessions) can help address a key barrier to rehabilitation compliance (i.e. expected dynamic pain) and hence help facilitate improved therapy compliance^9^.

Furthermore, this pilot study has allowed good insight into the possible complications from ambulatory CISB. Optimistically, the complications witnessed in this pilot study were all promptly detected, self-limiting and did not require additional treatment in any of the cases (aside from removal of catheter). For ambulatory CISB to be successfully deployed, there are several key learning points that can be gleaned from this study which can help optimise its use and minimise complications. The study team hopes to apply these learning points to future implementations of ambulatory CISB to utilise it in a safe and effective manner. Additionally, while the application of ambulatory CISB in such a setting and format is novel, the use of continuous nerve block catheters is not. The study team has also sought to contrast the learning points from this pilot study to existing literature on continuous nerve block catheters to improve the application of CISB for post-surgical analgesia for PHF patients.

Lesson 1: Patient selection is key. Stringent patient selection will reduce the risk of developing complications and minimise any health impacts to the patient should these complications occur. The study team proposes that the ambulatory CISB should only be considered in patients where the benefits are considerably greater than the risks. Patient factors (high pre-morbid functional demand such as those who are still employed or living independently, minimal co-morbidities) and surgeon factors (e.g. stability of fixation) should be regarded heavily.

Firstly, due to the location and nature of the interscalene nerve block, there is risk of ipsilateral phrenic nerve block and diaphragmatic hemiparesis resulting in a 25% reduction in pulmonary function – hence, ambulatory CISB should be contraindicated in any patient with respiratory insufficiency^16^. This learning point is reinforced by findings this study. Patient #5 experienced desaturation secondary to phrenic nerve palsy from the local anaesthetic infusion. This patient did well with supportive treatment as she did not have any underlying cardiorespiratory issues. While phrenic nerve palsy is an expected complication from CISB, good patient selection can ensure that such a complication (when they inevitably do arise), will be mild and self-limiting. Similarly, catheter-related complications such as erythema/infection were seen in a number of patients. Based on this, patients with co-morbidities that would predispose them to local infections due to an in-situ foreign body should ideally be excluded. This would include any condition that results in immunocompromise, such as poorly controlled diabetes. For example, of the 12 patients, only 2 patients in this study experienced local erythema at the CISB site – patient #2 who was likely predisposed to infection by his poorly controlled diabetes as well as patient #10 who had no co-morbidities but likely developed local erythema due to the prolonged duration of a foreign body being left in-situ – erythema developed on post-operative day 16 for her. In addition, as evidenced in patients #6 and #8, patient education and cooperation are paramount for the success of ambulatory CISB. Patients will need to be cognitively intact and be able to comply with post-operative instruction to care and manage the catheter at home.

Furthermore, ambulatory CISB should not be offered to every patient undergoing surgical management of PHF. Surgeon factors have to also be taken into consideration and the attending surgeon must be satisfied that the fixation/replacement is stable enough for early accelerated rehabilitation as facilitated by improved pain management from the CISB. Another aspect that can be further contemplated is if ambulatory CISB would give more favourable clinical outcomes for certain types of surgery. This is a retrospective finding discovered during data analysis and would be an interesting area to explore further in follow-up studies. In this study, limited statistical analysis showed that ambulatory CISB had significantly better dynamic pain control in RSA patients versus ORIF patients.

Lesson 2: Standardised Protocol to minimise complications (experienced anaesthesiologist, tunnelling technique), maximise analgesia efficiency (logistics, removal of catheter timing). For ambulatory CISB to be successful, there needs to be a standardised protocol to optimise its efficacy and reduce complications. Standardisation should be ensured on two fronts – procedurally and follow-up process.

In terms of procedure, there is a wealth of information and protocols to refer to within the current literature. Inconsistencies with the technique and inexperience can lead to increased incidence of complications. Instead, by learning from the experience of others, useful modifications can be made to existing protocol to improve the implementation of ambulatory CISB in our context. For instance, certain techniques used by other procedurists should be incorporated to improve precision/reduce risk of unintended nerve palsies (e.g. improving precision of catheter placement by stimulating the brachial plexus with the placement needle, performing the nerve block / catheter insertion under sedation, using ultrasound guidance to insert the catheters) or reduce risk of dislodgement (use of subcutaneous tunnelling)^17^. Within this study as well, the two dislodgements which occurred in non-tunneled catheters further reinforce this learning point. Once these dislodged catheters were replaced with tunneled catheters, no further dislodgment occurred. There other two dislodgements were likely due to inexperience with the technique, being the first CISB done by their respective procedurists in this study population. With greater experience and a standardisation in technique, such complications can be minimised.

Furthermore, through this pilot study, the study team has identified several logistical considerations that can help optimise efficiency/safety of ambulatory CISB: Firstly, the amount of local anaesthetic agent should be of sufficient quantity that it is able to cover all rehabilitation sessions until the next top-up but not too large such that it requires too large a balloon causing it to be a physical nuisance. Secondly, topping up of the balloon can be done when the patient comes for his/her rehabilitation session – this will help to minimise additional trips to the hospital for the patient. Thirdly, timing of removal should be pre-determined based on progress on rehabilitation. At the anticipated removal date, the LA dose can be calculated so that that the final rehabilitation session is done without any analgesia from the CISB. This will allow for a proper assessment of how much the pain interferes with the rehabilitation, and decision can then be made to see if the catheter needs to be continued or can be removed. In essence, if the patient tolerates session well, the catheter can be removed, but if there is still significant pain during rehabilitation, consideration for further LA top up can be considered. Lastly, there should be regular and close follow-up for these patients. Currently, there are daily video teleconsultations with the anaesthesia team as well as weekly to two-weekly clinic visits with both the anaesthesia and orthopaedic team. This together with serial radiographs of the affected shoulder will allow prompt detection of any complications and further management as necessary.

Lesson 3: Clinicians need to be familiar with the expected complications of CISB in order to utilise it safely as an analgesia option. The use of ambulatory CISBs in this population has also brought about the issue of diagnostic dilemmas, should any complications arise. For example, within this study, patient #9 experienced weak wrist extension which was initially attributed to nerve block from the CISB. Although this weakness was eventually attributed to radial nerve palsy from intra-operative neuropraxia (as symptoms did not improve with reduction of dose of LA / removal of CISB), this example has highlighted how the presence of the CISB may introduce new differential diagnoses to the equation if new symptoms arise in postoperative patients. The clinician needs to be aware of such potential differential diagnoses if he/she chooses to utilise CISB as an analgesia option – to be able to resolve any issues that may arise. Vitally, the ability to dial down the nerve block by reducing the infusion-rates of short-acting LA also allows us to determine if the neurological deficit is due to the nerve block or other factors.

Another important example is the case of patient #3, who experienced dyspnea and tachycardia post-operatively – while this patient’s symptoms were eventually attributed to a type 2 myocardial infarction due to intra and post-operative stressors, her initial presentation was extremely similar to patient #5 who suffered from phrenic nerve palsy from the nerve block. The clinician needs to be well-versed with the potential life-threatening complications that can arise from the CISB, such as how dyspnea post-CISB insertion can be a result from iatrogenic pneumothorax or phrenic nerve palsy, in order to safely utilise CISB as a novel analgesia option for post-surgical PHF patients. Familiarity with the expected complications of an in-situ CISB can also lend guidance to better patient selection – e.g. avoiding such a modality of analgesia in patients with poor cardiac/respiratory reserves.

For strengths and weaknesses, this pilot study was not without weakness, with a small sample size of 12 preventing any meaningful statistical analysis. Despite this, this pilot study has allowed a glimpse into the positive outcomes of ambulatory CISB in terms of satisfactory analgesia as well as largely self-limiting complications. The study team believes that this pilot has provided valuable insights and learning points into how ambulatory CISB can be optimised to minimise complications. This will serve as strong foundation for a well-designed large prospective trial to study ambulatory CISB to better understand the safety (complication rates) and efficacy (pain control, functional outcomes) between PHF patients with and without ambulatory CISB.

## Conclusion

This pilot study has shown us that this novel application of ambulatory CISB is a potentially safe and efficacious postoperative analgesia modality that should be studied further. The complications seen in this study have shown to be minor and self-limiting in most instances. In order to ensure that complications remain minor and are dealt with promptly should they arise, patient suitability (in terms of cognitive ability, comorbidities and surgical factors) must be considered. Further, standardised protocols (in terms of procedural and logistical considerations as well as routine nature of follow-up) and familiarity with the expected complications can allow ambulatory CISB to be utilised safely and effectively. Moving forwards, the study team plans on embarking on a prospective cohort study to evaluate the effectiveness and safety of ambulatory CISB, incorporating all the lessons learnt. Further analysis can also be performed to determine if ambulatory CISB does indeed provide superior pain control in arthroplasty patients versus open reduction internal fixation patients.
